# The association between attention deficit/hyperactivity disorder and internet addiction: a systematic review and meta-analysis

**DOI:** 10.1186/s12888-017-1408-x

**Published:** 2017-07-19

**Authors:** Bing-qian Wang, Nan-qi Yao, Xiang Zhou, Jian Liu, Zheng-tao Lv

**Affiliations:** 1 First Clinical College, Union Hospital, Tongji Medical College, Huazhong University of Science and Technology, Wuhan, Hubei China; 2 School of Public Health, Faculty of Medicine, University of Hong Kong, Hong Kong, China; 3 University of Heidelberg, Heidelberg, Germany; 4 Biological Engineering and Regenerative Medicine Center, Department of Orthopedics, Tongji Hospital, Tongji Medical College, Huazhong University of Science and Technology, 1095#, Jiefang Avenue, Qiaokou District, Wuhan, Hubei 430030 China; 5 Department of Orthopedics, Tongji Hospital, Tongji Medical College, Huazhong University of Science and Technology, 1095#, Jiefang Avenue, Qiaokou District, Wuhan, Hubei 430030 China

**Keywords:** Internet addiction, Attention-deficit/hyperactivity disorder, Meta-analysis, Systematic review

## Abstract

**Background:**

This study aimed to analyze the association between Attention Deficit/Hyperactivity Disorder (ADHD) and Internet addiction (IA).

**Methods:**

A systematic literature search was performed in four online databases in total including CENTRAL, EMBASE, PubMed and PsychINFO. Observational studies (case-control, cross-sectional and cohort studies) measuring the correlation between IA and ADHD were screened for eligibility. Two independent reviewers screened each article according to the predetermined inclusion criteria. A total of 15 studies (2 cohort studies and 13 cross-sectional studies) met our inclusion criteria and were included in the quantitative synthesis. Meta-analysis was conducted using RevMan 5.3 software.

**Results:**

A moderate association between IA and ADHD was found. Individuals with IA were associated with more severe symptoms of ADHD, including the combined total symptom score, inattention score and hyperactivity/impulsivity score. Males were associated with IA, whereas there was no significant correlation between age and IA.

**Conclusions:**

IA was positively associated with ADHD among adolescents and young adults. Clinicians and parents should pay more attention to the symptoms of ADHD in individuals with IA, and the monitoring of Internet use of patients suffering from ADHD is also necessary. Longitudinal studies controlling for baseline mental health are needed.

## Background

Internet addiction (IA), initially reported by Young [1], is considered as a new psychiatric disorder, but IA was still not listed as a clinical entity in the fifth edition of Diagnostic and Statistical Manual of Mental Disorders (DSM-5). People using Internet excessively and pathologically might suffer from adverse consequences, including arguments, fatigue, lying, poor grading in school or vocational achievement during working, social isolation and even functional problems such as school failure, job loss and marriage failure [2]. The pathway from adaptive Internet use to IA is very complicate and ambiguous, which could be affected by many different factors including both individual and environment. It’s reported that IA was prevalent in both eastern and western countries. Because of the different questionnaires, diagnostic criteria used, the prevalence of IA in different areas with different culture is different. It’s reported that the prevalence of IA ranges from 1% to 36.7% in a literature review [3]. Given the large scale of Internet usage and so many negative consequences, it is important to untangle the potential risks associated with IA.

In a previously published systematic review about the association between IA and Attention Deficit/Hyperactivity Disorder (ADHD), positive correlations were confirmed after controlling covariates [4]. A 2-year prospective study found that adolescents diagnosed as ADHD were the most likely to be addicted to the Internet than other psychiatric symptoms such as hostility and social phobia [5]. However, it remains a matter of debate that if there were indeed any causalities between IA and ADHD., and the association could be explained from different aspects. For instance, in the biopsychosocial model, “being easily bored” and “having an aversion for delayed rewards” are two main ADHD symptoms [6, 7]. Internet use provides multiple windows with a variety of activities at the same time and immediate reward may decrease the boredom feeling and reward quickly, which makes people with ADHD addicted to Internet more easily. Furthermore, some researchers also found that subjects with ADHD have abnormal brain activities that would lead to impaired inhibition, which results in lack of self-control ability, so that Internet users would become more unable to restrain themselves and vulnerable to IA. Thus, ADHD could be a possible risk factor that may lead to IA.

Two well-established systematic reviews have summarized relevant articles on the relation of IA and psychiatric comorbidities, but their conclusions regarding the association between IA and ADHD were hampered by the some methodological deficiencies and paucity of included studies, only five and four observational studies that reported odds ratio (OR) were included in two aforementioned studies respectively [4, 8]. The drawn conclusions were based upon ORs of unadjusted results, which could weaken the reliability of pooled results. As new evidence is emerging in recent years, it is necessary to perform an updated meta-analysis to reevaluate the association between IA and ADHD. Furthermore, our present study aims to assess the influence of IA on symptoms of ADHD, and to clarify the relationships between IA and demographic characteristics of enrolled participants.

## Methods

This systematic review was conducted in accordance to the Meta-analysis Of Observational Studies in Epidemiology (MOOSE) guidelines [9].

### Literature search

A comprehensive electronic literature search was conducted by using following databases: CENTRAL, EMBASE, PubMed and PsychINFO. Relevant articles published from inception to June 2016 were searched in databases above by two reviewers (B.Q. Wang and N.Q. Yao) independently and no language restriction was imposed. Free text words and Medical Subject Headings (MeSH) were employed as search terms independently or in combination of according to specifications of each database.

The following searching strategy was utilized: (Internet addiction or problematic Internet use or Internet addiction disorder or pathological Internet use or Internet game addiction or excessive Internet use or compulsive Internet use or Internet dependency or computer addiction) and (“Attention Deficit Disorder with Hyperactivity”[Mesh] or ADHD or ADDH or Attention Deficit Disorder with Hyperactivity or Attention Deficit-Hyperactivity Disorder or Hyperkinetic Syndrome or Attention Deficit Hyperactivity Disorder or Attention Deficit Disorder). The bibliographies of relevant systematic reviews and clinical guidelines were manually searched. References from each retrieved papers were also manually searched.

### Types of participants

Patients diagnosed with IA by a standard criterion were recruited in IA group. The tools employed for the assessment of IA included CIAS [10], IAT [11], IAS [12], along with other well-established tools. No restriction on age, race and gender was imposed.

### Types of control

Subjects without IA were included without other restrictions.

### Outcome measures

The primary outcome was adjusted odds ratio (AOR), secondary outcome measures included crude odds ratio (COR) and parameters assessing the severity of symptoms of ADHD. COR should be reported by included studies or could be calculated based upon raw data.

### Types of included studies

Observational studies including cohort studies, case-control studies and cross-sectional studies without restrictions on geographic area or sample size were included.

### Exclusion criteria

Case series, case repots, book chapters, editorials and papers of conferences were excluded. Studies failed to report the diagnostic criteria of IA were excluded. Studies on pathologic internet use such as spending time and time to sleep but without a specific definition of IA were also excluded. We also excluded case series, case reports and articles only studied the brain imaging, electroencephalogram (EEG), treatments, intervention or other related symptoms such as impulsivity, lifestyle and sexual attitude but not studying the relationship with ADHD. Studies with abstracts written in English language but with full-text in other languages were excluded. In addition, articles with only abstracts were also removed because detailed data could not be obtained so the methodologic quality of them could not be assessed.

### Data extraction

Two investigators (B.Q. Wang and N.Q. Yao) individually reviewed each article and were blinded to the process and outcomes of each other. According to the inclusion criteria defined above, we implemented a strict screening to include articles with the eligibility. Data was also collected independently from these selected articles using the same collection form including first author, country, year of the publication, study design, source of cases, sample size in each group, mean age of all the enrolled subjects, definition of IA and definition of ADHD, prevalence of ADHD in each group and scales used to assess the symptoms of ADHD. Any disagreement between the two reviewers was resolved through discussion until a consensus was reached. The third review author (Z.T. Lv) was consulted if an agreement could not be achieved.

### Methodological quality assessment

The Newcastle-Ottawa Scale (NOS) [13] and an adapted form of NOS [14] were utilized to assess the methodological quality of non-randomized studies in this systematic review. Two reviewers assessed the methodological qualities of each study independently, the results were compared afterwards.

### Data synthesis and analysis

OR and the associated 95% confidence interval (CI) in each included studies were combined in order to assess the possible association between IA and ADHD. The standardized mean difference (SMD) as well as the associated 95% CI was both calculated for severity of ADHD and combined using the same method. Prevalence of ADHD in IA groups was also combined, and stratified analysis was made by two age groups. As included studies measured the outcomes using different scales, the random-effect model was used to conduct the statistical analysis. Heterogeneity between studies was assessed by the Higgins I^2^ test (*P* > 0.1 and I^2^ < 50% indicate acceptable heterogeneity) and a standard chi-square test. And the heterogeneity outcomes showing *P* > 0.1 and I^2^ < 50% could be acceptable.

Meta-regression analyses on age (≥18 years and <18 years), ethnicity (Asian and European) and risk of bias (high, medium or low risk of bias) was implemented using Stata version 12.0 (Stata Corp LP, USA) to identify the probable cause of heterogeneity. Sensitivity analysis by removing each related study at a time was also made to evaluate the impact of each study on the pooled OR and the severity of ADHD symptoms. Begg’s rank correlation test and Egger’s linear regression test were used to evaluating the publication bias. The forest plot was made by RevMan 5.3 (Copenhagen: The Nordic Cochrane Centre, The Cochrane Collaboration, 2014).

The effect size of association was expressed as small, moderate and large according to Cohen. OR was converted into these groups according to Chinn [15]. Cohen’s d was calculated based on original data from studies that did not provide OR. Thus, the effect sizes were explained as follow: small (Cohen’s d = 0.2, OR = 1.44), moderate (Cohen’s d = 0.5, OR = 2.48) and large (Cohen’s d = 0.8, OR = 4.27).

## Results

### Literature search

**Fig. 1:**
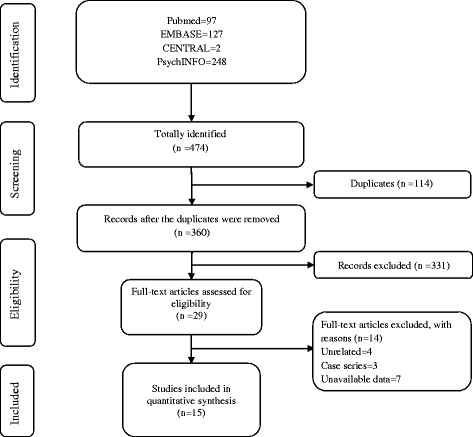
Flow chart of literature selection

Three hundred sixty studies were identified using the search strategy, including 2 from CENTRAL, 127 from EMBASE, 97 records from PubMed and 248 from PsychINFO (Fig. 1). After excluding 114 duplicated studies, a total of 360 articles were identified according to the present inclusion criteria. 29 potentially relevant studies were included in the systematic review and assessed by full-text. Among the remaining 29 articles, 3 case series were excluded, 4 studies were excluded because they were not associated with the relationship between IA and ADHD, 7 studies were removed because the outcome measures were unavailable. No additional studies were included through reference and bibliographic review. Finally, 15 studies were deemed eligible for the meta-analysis.

### Main characteristics of included studies

Two cohort studies [16] and thirteen cross-sectional studies published from 2004 to 2016 were identified and included in our current study. The majority of studies were conducted in Taiwan [16–21] and South Korea [22–24], the remaining studies were performed in Turkey [25–27], Sweden [28] and in the US [29]. Targeted population were either adolescents or young adults, both genders were evaluated in all studies. The prevalence of ADHD in IA groups ranged from 19.5% to 42.5%, while the prevalence of ADHD in control groups ranged from 4.6% to 15.2%. Various scales or questionnaires were employed for the assessment of IA: CIAS [10], CIAS-R [30], IAT [11], IAS [12], BAPINT-SV [31], PRIUSS [32], DC-IA-C [33], GAIT [34] and DSM-5; and ADHD: SNAP-IV [35], ASRS [36], CASS: short [37], CASS [37], K-ARS [38], ADHDS [39] and DSM-IV-TR. Main characteristics of included studies were summarized in Table 1.

**Table 1:** Main characteristics of included studies

Study	Study design	Sample size (IA/control)	Source of IA cases	Mean age of subjects (years)	Definition of IA	Definition of ADHD	Prevalence of ADHD	Symptom severity of ADHD
Chen, 2015Taiwan	Cohort study	131/1022	grade 3 and 5 and grade 8 students in Northern Taiwan	N.R.	CIAS	SNAP-IV	N.R.	SNAP-IV (inattention, Hyp-Imp)
Cheng, 2014Taiwan	Cross-sectional	339/1282	incoming students at National Cheng Kung University	N.R.	CIAS-R	ASRS	IA:42.5%control:15.2%	N.R.
Cho, 2008South Korea	Cross-sectional	125/561	child and adolescent psychiatric outpatient clinics of two medical centers	N.R.	IAT	CASS: short	N.R.	CASS
Dalbudak, 2014Turkey	Cross-sectional	159/112	students from Turgut Ozal University	N.R.	IAS	ASRS	N.R.	ASRS(inattention, Hyp-Imp)
Dalbudak, 2015Turkey	Cross-sectional	64/518	from two universities	20.99	BAPINT-SV	ASRS	N.R.	ASRS (inattention, Hyp-Imp)
Hyun, 2015South Korea	Cross-sectional	255/153	who visited the Online Game Clinic Center at OO University Hospital	20.69	CIAS	K-ARS	N.R.	K-ARS
Jelenchick, 2014the US	Cross-sectional	N.R.	older adolescents aged 18 to 25 years from a nutritional sciences course at a public university	19.7	PRIUSS	ASRS	N.R.	N.R.
Ko, 2008Taiwan	Cross-sectional	87/129	respondents to an advertisement regarding internet usage	21.5	DC-IA-C	semi-structured Diagnostic Tool based on the DSM-IV	IA:32.2% control:8.5%	N.R.
Ko, 2009Taiwan	Cohort study	276/1572	7th grade students from 10 junior high schools	12.4	CIAS	ADHDS	IA:19.5%control:10.1%	N.R.
Metin, 2015Turkey	Cross-sectional	61/710	students from three different high schools	16.9	CIAS	Adult ADD/ADHD Diagnostic and Assessment Inventory based on the DSM-IV	IA:36.1%control:9.6%	Adult ADD/ADHD Diagnostic and Assessment Inventory
Sofia, 2016Sweden	Cross-sectional	N.R.	from the child and adolescent psychiatric clinics, or community sample	14.07	GAIT	ASRS-A	N.R.	N.R.
Yen, 2007Taiwan	Cross-sectional	338/1552	3 of 33 senior high schools, and 7 of 20 vocational high schools	16.26	CIAS	ADHDS	N.R.	ADHDS
Yen, 2009Taiwan	Cross-sectional	338/2281	students from 8 colleges	20.46	CIAS	ASRS	IA:20.7%control:8.3%	ASRS (inattention, Hyp-Imp)
Yen, 2016Taiwan	Cross-sectional	87/87	advertisements in University campuses and bulletin board systems	23.34	DSM-5	DSM-IV-TR	IA:39.1%control:4.6%	N.R.
Yoo, 2004South Korea	Cross-sectional	80/455	elementary school students	11.1	IAT	K-ARS	IA:22.5%control:8.1%	K-ARS (inattention, Hyp-Imp)

*IA* Internet addiction; *ADHD* attention-deficit/hyperactivity disorder; *Hyp*-*Imp*: hyperactivity-impulsivity; *CIAS* Chen Internet Addiction Scale; *CIAS*-*R* Chen Internet Addiction Scale-Revision; *GAIT* Gaming Addiction Identification Test; *SNAP*-*IV* the Swanson, Nolan, and Pelham IV; *ASRS* Adult ADHD Self-Report Scale; *CASS* Short: Conners/Wells Adolescent Self-Report Scale: Short Form; *CASS* Conners/Wells Adolescent Self-Report of Symptoms; *IAS* Internet Addiction Scale; *IAT* Internet Addiction Test; *BAPINT*-*SV* Addiction Profile Index Internet Addiction Form Screening Version; *K-ARS* Korean version of DuPau’s ADHD rating scale (K-ARS); *PRIUSS* Problematic and Risky Internet Use Screening Scale; *DC*-*IA*-*C* Diagnostic Criteria of Internet Addiction for College Students; *ADHDS* Attention-Deficit /Hyperactivity Disorder Self-Rated Scale; *DSM*-*5* Diagnostic and statistical manual of mental disorder (5th edition); *DSM*-*IV* Diagnostic and statistical manual of mental disorder (4th edition); *DSM*-*IV*-*TR* Diagnostic and statistical manual of mental disorder (4th edition)(text revision); *N*.*R*. not reported

### Methodological quality

The NOS scale was used to assess the methodological quality in cohort studies, the adapted form of the NOS was utilized for the assessment of cross-sectional studies. Studies were categorized into low (scored 8–9), medium (scored 6–7), and high risk of bias groups (scored ≤5). 8 studies [16–18, 21, 23, 27–29] were judged to high risk of bias, the remaining 7 studies [19, 20, 22, 24–26] were medium risk of bias. The detailed information about methodological quality assessment was presented in Tables 2 and 3.

**Table 2:** Methodological quality of cohort studies

Item	Chen, 2015	Ko, 2009
Representativeness of the exposed cohort	-	*
Selection of the non-exposed cohort	*	*
Ascertainment of exposure	*	*
Demonstration that outcome of interest was not present at start of study	-	-
Comparability of cohorts on the basis of the design or analysis	--	--
Assessment of outcome	*	*
Was follow-up long enough for outcomes to occur	*	*
Adequacy of follow up of cohorts	*	*

A study could be awarded a maximum of one star for each item except for the item Comparability of cohorts on the basis of the design or analysis

**Table 3:** Methodological quality of cross-sectional studies

Study	Representative-ness of the sample	Sample size	Non-respondents	Ascertainment of the exposure	Comparability	Assessment of the outcome	Statistical test
Cheng, 2014	-	*	-	**	--	*	*
Cho, 2008	*	*	-	**	--	*	*
Dalbudak, 2014	*	*	-	**	--	*	*
Dalbudak, 2015	*	*	-	**	--	*	*
Hyun, 2015	-	*	*	**	--	*	*
Jelenchick, 2014	-	*	-	**	--	*	*
Ko, 2008	-	*	-	**	--	*	*
Metin, 2015	-	*	-	**	--	*	*
Sofia, 2016	-	*	-	**	--	*	*
Yen, 2007	*	*	*	**	--	*	*
Yen, 2009	*	*	-	**	--	*	*
Yen, 2016	-	*	-	**	--	*	*
Yoo, 2004	-	*	-	**	--	*	*

A study could be awarded a maximum of one star for each item except for the item Comparability

### Evidence of association between IA and ADHD

**Fig. 2:**
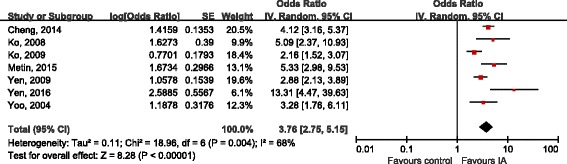
Forest plot of crude OR

**Fig. 3:**
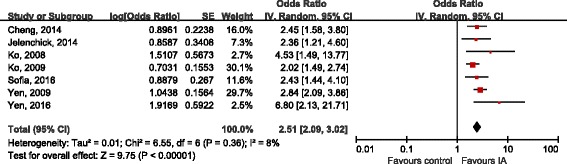
Forest plot of adjusted OR

Seven studies [17, 18, 20, 21, 23, 27] reported COR as outcome, 7 studies [17, 18, 20, 21, 28, 29] calculated AOR after adjusting for potential confounders (gender, age, school bullying, family maltreatment, race, educational level, lifestyle factors, physical factors, mental factors and social factors). All these studies showed a consistency regarding the presence and direction of association, the prevalence of ADHD was found to be higher in IA subjects than in non-IA subjects amidst the selected study. The combination of COR showed a statistically significant correlation between IA and ADHD (OR 3.76, 95%CI 2.75, 5.15; Tau^2^ = 0.11, Chi^2^ = 18.96; df = 6 (*P* = 0.004), I^2^ = 68%), obvious heterogeneity between studies existed (Fig. 2). After controlling potential confounding factors, the pooled AOR indicated that patients with IA were 2.51 times more likely to be diagnosed with ADHD when compared with non-IA subjects (OR 2.51, 95%CI 2.09, 3.02; Tau^2^ = 0.01, Chi^2^ = 6.55; df = 6 (*P* = 0.36), I^2^ = 8%), the heterogeneity across related studies was low (Fig. 3). The effect sizes were shown in Table 4, the association between IA and ADHD were small [17, 28, 29], moderate [20, 23, 25] or large [16, 18, 19, 21, 22, 24, 26, 27].

**Table 4:** Estimated effect sizes of included studies

Study	OR	Cohen’s d	Effect size
Chen, 2015	-	1.86	Large
Cheng, 2014	2.45	-	Small
Cho, 2008	-	1.17	Large
Dalbudak, 2014	-	0.76	Moderate
Dalbudak, 2015	-	0.83	Large
Hyun, 2015	-	1.11	Large
Jelenchick, 2014	2.36	-	Small
Ko, 2008	4.53	-	Large
Ko, 2009	2.02	-	Small
Metin, 2015	-	1.03	Large
Sofia, 2016	2.43	-	Small
Yen, 2007	-	0.83	Large
Yen, 2009	2.84	-	Moderate
Yen, 2016	6.80	-	Large
Yoo, 2004	-	0.69	Moderate

*OR* odds ratio; Large: Cohen’s d = 0.8, OR = 4.27; Moderate: Cohen’s d = 0.5, OR = 2.48; Small: Cohen’s d = 0.2, OR = 1.44

### Age and IA

Seven studies [17, 18, 20, 21, 23, 27] reported prevalence of ADHD in IA groups. The combined prevalence showed that prevalence of ADHD in different age groups were similar: <18 years (prevalence 0.25, 95%CI 0.16, 0.33; Tau^2^ = 0, Chi^2^ = 6.24, df = 2 (*P* = 0.04), I^2^ = 68%), ≥18 years (prevalence 0.23, 95%CI 0.08, 0.39; Tau^2^ = 0.02, Chi^2^ = 116.15, df = 2 (*P* < 0.00001), I^2^ = 97%).

Among our selected studies, 7 [16, 19, 22, 23, 27, 28] targeted adolescents and the remaining 8 studies [17, 18, 20, 21, 24–26, 29] targeted young adults. The effect size of these studies were also similar, adolescents: 4 large, 1 moderate and 2 small; young adults: 4 large, 2 moderate and 2 small. In addition, 6 of our included studies determined the association between age and IA, no study reported a statistically significant association between age and IA after controlling confounding factors.

### Gender and IA

6 studies [16, 18, 19, 21, 28] reported significant gender difference, the prevalence of IA was significantly higher in male subjects than female. No study found higher rate of IA in females.

### IA and symptoms of ADHD

**Fig. 4:**
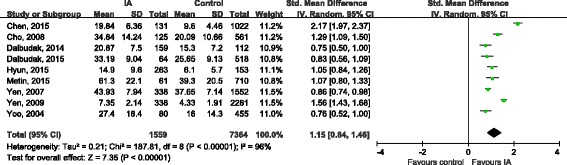
Forest plot of total symptom score

**Fig. 5:**
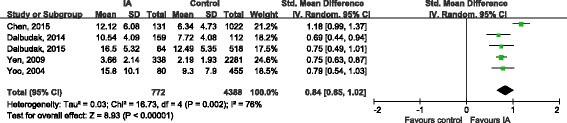
Forest plot of inattention score

**Fig. 6:**
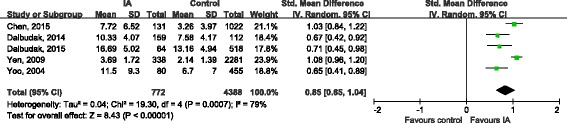
Forest plot of hyperactivity/impulsivity score

Nine studies [16, 19, 21–27] evaluated severity of symptoms in ADHD using series of scales. The combination of total score showed that the overall severity of symptoms of ADHD in IA groups were significantly worse than healthy control (SMD 1.15, 95%CI 0.84, 1.46; Tau^2^ = 0.21; Chi^2^ = 187.81, df = 8 (*P* < 0.00001); I^2^ = 96%) (Fig. 4). The symptoms of inattention (SMD 0.84, 95%CI 0.65, 1.02; Tau^2^ = 0.03; Chi^2^ = 16.73, df = 4 (*P* = 0.002); I^2^ = 76%) and hyperactivity/impulsivity (SMD 0.85, 95%CI 0.65, 1.04; Tau^2^ = 0.04; Chi^2^ = 19.30, df = 4 (*P* = 0.0007); I^2^ = 79%) in IA groups were also significantly more serious than that in health control groups (Figs. 5 and 6).

### Meta-regression and sensitivity analysis

Meta-regression was conducted by residual (restricted) maximum likelihood (REML) with Knapp-Hartung modification to find the potentially possible source of heterogeneity in severity of symptoms of ADHD, the results of meta-regression by age, ethnicity and risk of bias were presented in Table 5. However, neither age pattern nor ethnicity was not significantly associated with the heterogeneity between studies. Risk of bias of included studies could be a potential source of heterogeneity in the total symptom score and severity of hyperactivity-impulsivity (Table 5).

**Table 5:** Metaregression of basic characteristics of trials and severity of symptoms in ADHD

Outcome	No. of studies	Factor tested	P	Adjusted R^2^
Total symptom score	9	age	0.296	2.69%
		ethnicity	0.254	6.74%
		risk of bias	0.026	49.92%
Severity of inattention	5	age	0.16	54.94%
		ethnicity	0.421	−5.75%
		risk of bias	0.345	0.80%
Severity of Hyp-Imp	5	age	0.972	−40.99%
		ethnicity	0.257	29.91%
		risk of bias	0.022	100.00%

*ADHD* attention-deficit/hyperactivity disorder; *Hyp*-*Imp* hyperactivity-impulsivity

In the severity of symptoms ADHD, studies with high risk of bias (SMD 1.60, 95%CI 1.07, 2.13; Tau^2^ = 0.21; Chi^2^ = 46.11, df = 2 (*P* < 0.00001); I^2^ = 96%) had significantly higher total score when compared with studies with medium risk of bias (SMD 0.93, 95%CI 0.77, 1.09; Tau^2^ = 0.03; Chi^2^ = 18.84, df = 5 (*P* = 0.002); I^2^ = 73%). In the severity of hyperactivity/impulsivity, studies with high risk of bias (SMD 1.06, 95%CI 0.96, 1.16; Tau^2^ = 0.00; Chi^2^ = 0.20, df = 1 (*P* = 0.66); I^2^ = 0%) had significantly higher score than studies with medium risk of bias (SMD 0.67, 95%CI 0.53, 0.82; Tau^2^ = 0.00; Chi^2^ = 0.13, df = 2 (*P* = 0.94); I^2^ = 0%). When studies with high risk of bias were removed from meta-analyses, the overall symptom and hyperactivity/impulsivity in IA groups were still significantly more serious than that in health control groups. Sensitivity analysis contributed to the stability of resulting effects (detailed data not shown).

### Publication bias

Publication bias was detected using Begg’s rank correlation test and Egger’s linear regression test, the results were shown in Table 6. A publication bias in the severity of hyperactivity/impulsivity was found.

**Table 6:** Publication bias of outcomes

Outcome	Begg’s test		Egger’s test	
	z	P	t	P
COR	0.9	0.368	1.98	0.116
AOR	1.5	0.133	2.24	0.075
Total symptom score	0.31	0.754	−0.46	0.66
Severity of inattention	−0.24	1	0.18	0.868
Severity of Hyp-Imp	0.73	0.462	−4.22	0.024

*COR* crude odds ratio; *AOR* adjusted odds ratio; *Hyp*-*Imp* hyperactivity-impulsivity

## Discussion

In summary, the finding of our present study suggested a positive association between IA and ADHD even after controlling confounding factors, symptoms of ADHD in IA groups were more severe than control groups. Male adolescents and young adults were more likely to be diagnosed with IA, but age pattern was not positively associated with IA in our included studies. Evidence support a causal relation between IA and ADHD is still lacking.

To the best of our knowledge, this is the first meta-analysis to individually investigate the association between IA and ADHD with consideration of heterogeneity while the previously published articles were systematic reviews about IA and several psychiatric co-morbidities [4, 8] or narrative literature review about the association between Internet gaming disorder and ADHD [40]. In two previously published systematic reviews, only few studies reporting OR as outcome were included for meta-analysis. The extracted ORs were pooled without differentiating COR and AOR, which might lead to an exaggeration or underestimation of the correlations between IA and ADHD. Thus, the conclusions drawn by aforementioned studies should be interpreted with caution. The results of our current work were powered by sufficient number of included studies and rigorous methodological quality assessment by independent reviewers. Both dichotomous variables and continuous variables were taken into consideration by our study, which greatly fill the blank of now-existing literature. In addition, the magnitude of effect size across studies was compared according to Cohen.

ADHD patients have poor self-control ability, so they’re more easily to sustain an addiction to substances as well as Internet. But studies have reported that striatal dopamine could help game users focus and gain better performance while playing Internet games [41], which let ADHD patients compensate for the failure in real-life and prefer into the virtual world. Compared with real life, Internet users would get response, reward and establish interpersonal relationships more easily online. Our results demonstrated that patients with IA were present with more severe symptoms of ADHD than healthy control, so that IA may also have influence on ADHD. Ko and colleagues reported that ADHD could predict the occurrence of IA in the 2-year follow-up. Chen et al. [16] also reported that high ADHD symptoms were significantly associated with the occurrence of IA. In summary, IA and ADHD may interact with each other. However, evidence supporting a causality between IA and ADHD is still lacking, only two included studies were based on a prospective design. The causality between both entities is still a matter of debate.

The majority of our included studies suggested moderate and strong associations between IA and ADHD. The obviously observed heterogeneity (I^2^ = 68%) in combination of COR suggested that demographic factors and other social or family factors could possibly affect the association, this hypothesis was partially verified by the low heterogeneity in pooled AOR (I^2^ = 8%). We further undertook meta-regression by age, ethnicity and methodological quality to determine whether they contributed to the heterogeneity in symptoms score, methodological quality of included studies was found to be associated with heterogeneity across studies. Thus, prospective cohort studies with high methodological quality are required.

The risk of addiction to Internet was higher among males than females, which might be explained by two reasons. Firstly, more males than females tend to seek self-esteem feelings and make social contacts online. Secondly, girls may receive more close supervision regarding internet use than boys in a family. Studies found that inattention was the most associated symptoms of ADHD among young adults and more significant in female adults.

Except for gender difference, other factors for IA such as low family support, protective parenting style, poor grading in school, bad interpersonal ability [42] might predict IA. These predicting factors found could be specifically targeted when designing the prevention program for IA among the children and adolescent population. On the other hand, these factors might be confounders that could affect the association between IA and ADHD, which should be controlled in more prospective cohort studies in the future. Studies showed that 65% children having ADHD during childhood had persistent ADHD symptoms till their adulthood [43]. Adult ADHD brings many negative effects but is seldom known by public [44]. Thus, ADHD patients should be very significant target group for the prevention for IA.

An interesting phenomenon observed in our study is that most included studies were performed in Asian countries. It was reported in a study of Zhang et al. [45] that IA was more prevalent in some Asian countries than in the United States. A possible reason might be the differences in sociocultural background [46]. Unlike in Asia, where Internet cafés are easily accessible and frequently used, in the US games and virtual sex are accessed from the home. Furthermore, attempts to evaluate the phenomenon are impeded by shame, denial, and minimization [47]. However, this explanation should be confirmed in further studies.

Given the results found in the current study, problems of IA, ADHD as well as the comorbidity conditions are required to get more concern of public health. Government should look for effective prevention policies and strategies to reduce the related health risks and negative outcomes. First, IA was identified as an emerging public health issue in both South Korea and China, as well as Taiwan. But until now, the scope of a universal definition of IA is still absent. A standard terminology as well as diagnostic criterion should be established and the cross-cultural validity should be examined to enable international comparisons. Secondly, according to the results of our current study, age was not associated with IA, which suggested that both adolescents and young adults should be targeted for the IA prevention. Last but not least, until now, although the significant relationship between IA and ADHD was proved, whether the ADHD is the risk factor of IA or a comorbidity is still unknown. It’s suggested that the comorbidity disease should be cautiously screened if diagnosed one of them. ADHD symptoms should be carefully prevented and early identified among at-risk subjects and their families by effective strategies.

There were several limitations in our study. Firstly, only two prospective cohort studies conducted in Taiwan were included in our study [16], definite conclusion about the causal relationship between ADHD and IA could not be drawn. Secondly, homogeneous geographic distribution and lack of a universal diagnostic criterion for IA are two primary problems still remaining. The prevalence of IA is inconsistent across included studies, except for cultural reasons and sample selection, the varying questionnaires and thresholds employed may contribute to the variations in these results. Seven of our included studies used CIAS criteria to diagnose IA with the cut-off point of 64 (accuracy 87.6%, specificity 92.6%). But to what extend could different questionnaires and thresholds employed influence the prevalence of IA could not be addressed by our current study. More studies need to be carried out in other geographic areas of the world for comparisons among different cultures, when using unified diagnostic criteria. Lastly, many of our included studies had recruitment bias because the method of sampling was highly selective, the conclusions could not be generalized to community population. Within the fifteen included studies, eight were evaluated with high risk of bias and seven had medium risk of bias. Thus the results should be interpreted cautiously.

## Conclusion

Taken together, our results demonstrated a moderate association between IA and ADHD in adolescents and young adults, patients with IA were present with more severe symptoms of ADHD than healthy control. More attention should be paid by parents and clinicians to patients with Internet addiction, and the symptoms of ADHD should be carefully evaluated. To address the causality between IA and ADHD, topic about whether treating ADHD could affect the Internet use behaviors should be systematically evaluated. On the other hand, future prospective cohort studies are encouraged to investigate whether treating IA would benefit or deteriorate the severity of ADHD.

## Abbreviations

ADDH: Attention Deficit Disorder with Hyperactivity
ADHD: Attention Deficit/Hyperactivity Disorder
AOR: Adjusted odds ratio
CI: Confidence interval
COR: Crude odds ratio
DSM-5: the fifth edition of Diagnostic and Statistical Manual of Mental Disorders
EEG: Electroencephalogram
IA: Internet addiction
MeSH: Medical Subject Heading
MOOSE: Meta-Analysis of Observational Studies in Epidemiology
NOS: Newcastle-Ottawa Scale
OR: Odds ratio
REML: Residual (restricted) maximum likelihood
SMD: Standardized mean difference
